# Study on the Evolution Behavior of Humidity Fields in Cement Concrete Pavements of a Coastal Airport During Early Stages in Humid and Hot Areas

**DOI:** 10.3390/ma16165643

**Published:** 2023-08-16

**Authors:** Min Chai, Changbin Hu, Lijuan Wang, Tao Chen

**Affiliations:** 1 School of Civil Engineering, Fuzhou University, Fuzhou 350108, China; 2 Fujian Jiangxia University, Fuzhou 350108, China

**Keywords:** cement concrete pavement, humid and hot climate, early stage, humidity field

## Abstract

Airport pavements are prone to early defects during the construction phase, and their early performance during the construction phase is significantly affected by the external temperature field. This article takes the concrete pavement of Xiamen Xiang’an New Airport as an example and uses a three-dimensional (3D) humidity simulation program for cement concrete pavement to study the evolution behavior of the early stage humidity field of the pavement in a humid and hot climate environment. The results indicate that the evolution law of the humidity field of the concrete pavement slab was consistent with the environmental field, presenting a 24 h periodic variation. The environmental field had a significant impact on the humidity of the surface layer of the pavement slab, and the humidity decreased rapidly with time. There was a humidity gradient on both the horizontal plane and the cutting plane of the slab, the horizontal humidity was concentrated from the boundary into the middle of the slab, and the sectional humidity was concentrated from the top to the bottom of the slab. Environmental parameters, construction parameters, and material and structural parameters all influenced humidity through humidity exchange or by changing the saturated vapor pressure inside the slab. The humidity field was most sensitive to environmental humidity and maintenance methods, but less sensitive to material parameters and structural parameters. Through analysis, it is advisable to avoid hot seasons, choose periods of increased environmental humidity, adopt appropriate maintenance methods during construction to reduce humidity stress on the slab, and therefore decrease early stage deterioration and improve service life.

## 1. Introduction

Under environmental loads, the volume deformation of road slabs due to temperature and humidity differences affects the service performance of cement concrete. The runway of the studied coastal airport in a humid and hot area is located in a coastal environment with typical high temperatures, high humidity, and strong winds. Previous research demonstrated that the runway is even more severely damaged by the natural environment than by wheel loads. The early performance of cement concrete pavement slabs during the construction phase is significantly affected by the external temperature field. Under the impact of a certain temperature and humidity field, the pavement slabs could crack due to excessive shrinkage stress and are extremely prone to the occurrence of early construction defects during the construction stage [1]. Thus, how to avoid early defects and ensure quality has been a key challenge for airport pavement slab engineering. Targeted research on the relationship between the meteorological environment and early defects is a key technology in proving that quality is a key aspect of airport pavement engineering.

The temperature difference and its impact on cement concrete pavement have been studied extensively [1,2,3,4,5,6,7]. In the code for design of pavement slab in China, the influence of temperature difference has also been taken into account regarding the design and calculation of pavement slabs through empirical values in different regions. Compared to the influence of temperature fields, the study concerning the influence of humidity fields was insufficient, and humidity fields are not considered in the current concrete pavement design and calculation [8]. Nevertheless, with the continuous deepening of research on the impact of early stage behaviors, it has been recognized that humidity had a significant impact on the deformation and stress of the pavement slabs [9,10,11,12,13,14,15,16,17,18,19,20]. Rania E. Asbahan et al. [21] measured the curvature of concrete slabs caused by humidity strain after 1–2 years through experiments. Wei, Qin, et al. [22] carried out numerical simulation work, and it was found that under extreme dry environments, the humidity warping order of the pavement slabs reached 2.52 mm, which was equivalent to the daily temperature difference. In addition, a relatively large humidity difference causes great humidity shear stress on the surface of the concrete pavement slabs, which leads to the delamination of the pavement slabs and develops into early an peeling phenomenon at the edges and corners of the slabs [23].

Unfortunately, the research on humidity fields has mainly focused on a two-dimensional (2D) perspective, and the impact of the diffusion effect on humidity fields could not be considered in a multidimensional manner. Moreover, there has been a lack of understanding of the behavior of the humidity evolution process of concrete pavement slabs from the curing period to the service period, and comparative research on humidity fields in different regions has also rarely been reported. Based on the above issues, the present paper intends to research the three-dimensional (3D) humidity field characteristics of the slabs at an early stage through 3D humidity numerical simulation. Research has shown that the environmental field has a significant impact on the humidity of concrete panels, with the horizontal direction having the greatest impact on the slab corners and the smallest impact on the slab corners. The humidity at the bottom of the slab in the direction of slab thickness is greater than that at the top of the slab, and the humidity difference varies periodically with the environment. This study can provide theoretical support for further improving pavement performance.

## 2. Field Simulation Program 3D Humidity

### 2.1. Calculation Theory of 3D Humidity Field

#### 2.1.1. Field Calculation Model of 3D Humidity

In a certain area of concrete, the internal moisture variation under the influence of humidity diffusion and cement hydration can be expressed as

(1) $$∆W=∆W_{d}+∆W_{s}$$

In the equation, ∆*W* is the total moisture variation, ∆*W_d_* is the moisture variation caused by diffusion, and ∆*Ws* is the self-drying moisture variation caused by cement hydration.

Due to the fact that the internal moisture of concrete is in a thermodynamic equilibrium state, it can be explained based on the principles of adsorption and desorption. The variation of in the internal relative humidity of concrete (∆*h*) is approximately linearly related to the variation in moisture content (∆*W*). After writing Equation (1) as a differential equation, it can be transformed into

(2) $$\frac{\partial h}{\partial t}=\frac{\partial h_{d}}{\partial t}+\frac{\partial h_{s}}{\partial t}+\kappa \frac{\partial T}{\partial t}$$

In the equation, ∂h/∂t is the total relative humidity variation in the slab, ∂hd/∂t is the humidity variation caused by diffusion, ∂hs/∂t is the relative humidity variation caused by self-drying, and κ∂T/∂t is the variation value of the relative humidity under the influence of temperature.

The expressions and differential equations of the three components in Equation (2) are as follows:

(1) Humidity diffusion model

**Figure 1:**
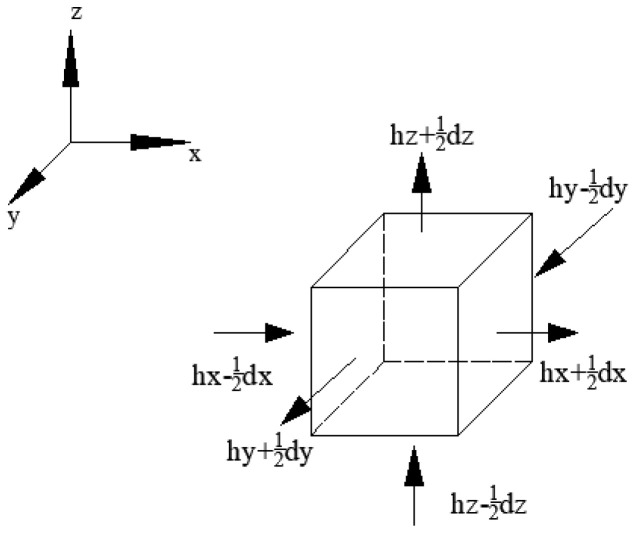
Humidity diffusion model.

Researchers conducted numerical research on humidity diffusion based on Fick’s law and mass conservation law [24]. As shown in Figure 1, the transmission speed of the diffusing substances per unit area in a certain region is proportional to the concentration gradient in that region, and Equation (2) could be converted into:

(3) $$\frac{\partial (h-h_{s}-\kappa \cdot \partial T)}{\partial t}=div\left(D\cdot grad\cdot h_{d}\right)$$

Let $h_{d}=h-h_{s}-\kappa \cdot \partial T$, and the following formula can be obtained:

(4) $$\frac{\partial h_{d}}{\partial t}=div\left(D\cdot grad\cdot h_{d}\right)$$

In the equation, the humidity diffusion coefficient D is a function of h [25], which can be expressed as

(5) D(h)=D0α0+1−α01+1−h/1−hc0n

In the equation, $D_{0}$ is the humidity diffusion coefficient of saturated concrete; *n* is the regression coefficient of the nonlinear diffusion equation of humidity, representing the declining rate of $D\left(h\right)$, and the value usually taken as 6~16; $\alpha_{0}=D_{\min}/D_{0}$, and $\alpha_{0}=D_{\min}/D_{0}$ is the diffusion coefficient when h=0; $h_{c0}$ is the relative humidity value when D is $(D_{0}-D_{\min})/2$, and the value is usually taken as 0.75~0.8.

(2) Temperature correction model

When the moisture content in the pores remains unchanged, the saturated vapor pressure changes with temperature, indirectly affecting the relative humidity h and the humidity diffusion coefficient $D_{0}$ of saturated concrete. The effect of temperature on $D_{0}$ and h can be expressed by Equation (6) [21]:

(6) $$\kappa =0.0135h\left(1-h\right)/\left(1.25-h\right)$$

(7) $$\frac{D_{T}}{D_{0}}=\frac{T}{T_{0}}\exp\left(\frac{Q}{RT}-\frac{Q}{RT_{0}}\right)$$

In the equation, κ is the humidity diffusion coefficient, T is the absolute temperature (°K); $T_{0}$ is the reference temperature, and room temperature is usually selected, and $D_{T}$ and $D_{0}$ are the saturated diffusion coefficient of concrete at T and $T_{0}$, respectively; Q is the activation energy of hydration.

(3) Self-drying model

The relationship between the relative humidity variation $∆h_{as}$ caused by self-drying and the hydration degree α can be expressed by Equation (8) as follows:

(8) $$∆h_{as}=\frac{a+\alpha }{b}$$

In the equation, $∆h_{as}$ is the relative humidity variation caused by self-drying; α is the hydration degree; a, b is a shape parameter; a=0.1, b=10 was adopted from the reference.

The hydration degree α at the equivalent time of the exponential function model was adopted; the formula is as follows:

(9) $$\alpha \left(t_{e}\right)=\alpha_{u}\cdot \exp\left[-{\left(\frac{\tau }{t_{e}}\right)}^{\beta }\right]$$

In the equation, $t_{e}$ is the equivalent time (h), $\alpha \left(t_{e}\right)$ is the hydration degree at $t_{e}$; $\alpha_{u}$ is the final hydration degree; τ is the hydration time parameter (h); β is the hydration shape parameter, and the specific values of τ and β are shown in the literature [26].

#### 2.1.2. The Determining Solution Conditions of 3D Humidity Diffusion Differential Equation

The boundary model between the initial humidity value and the moisture exchange between the internal and external is a key factor in determining humidity diffusion. The conditions of the selected initial humidity value and boundary conditions are as follows:

(1) Initial moment

The concrete is defaulted to be fully saturated when it is first formed, and the humidity at the initial moment is

(10) h(x,y,z,0)=1,(t=0)

(2) Surface boundary conditions

The water exchange between the environment and concrete is a complicated process, and it is difficult to calculate using pure theoretical models. The concrete evaporation rate model obtained by Al-Fadhala and Hover [27] was selected to calculate the water exchange with the environment.

(11) $$E_{r}=E_{W}\cdot \exp\left(-{\left(\frac{t}{a}\right)}^{1.5}\right)$$

In the equation, $E_{r}$ is the evaporation rate of water on the concrete surface; $E_{W}$ is the evaporation rate of water from the free water surface; t is time; a is a time constant. Generally, 3.75 h is taken for concrete and 6.16 h for mortar.

The formula of the free water surface evaporation rate proposed by Paul was selected:

(12) $$E_{W}=0.313\left(e_{so}-h\cdot e_{sa}\right)\cdot \left(0.253+0.06w\right)$$

In Equation (12), h is the relative humidity; $e_{so}$ is the vapor pressure on the concrete surface, kPa; $e_{sa}$ is the vapor pressure of air, kPa; w is the windspeed, km/h; and $e_{so}$ and $e_{sa}$ can be obtained from the following equation:

(13) $$e_{s}=0.61\exp\frac{17.3T}{273.3+T}$$

In Equation (13), T is the temperature of concrete or air, °C.

(3) Lower surface boundary conditions

Assuming that the lower surface of the concrete is in a sealed state without humidity exchange, the boundary conditions are

(14) $$\frac{\partial h}{\partial z}=0,z=l,t>0$$

#### 2.1.3. Diffusion Difference Derivation of 3D Humidity Field

The unit water diffusion modes at different locations within the slab are different. The units were divided into six types based on the number of diffusion surfaces. A 3D humidity diffusion equation was established as an example using a six-sided diffusion unit of 3D diffusion. The 3D differential format is

(15) $$\frac{\partial h}{\partial t}=\frac{\partial }{\partial x}\left(D\cdot \frac{\partial h}{\partial x}\right)+\frac{\partial }{\partial y}\left(D\cdot \frac{\partial h}{\partial y}\right)+\frac{\partial }{\partial z}\left(D\cdot \frac{\partial h}{\partial z}\right)$$

The equation was listed at the initial node of the period, the forward difference quotient was taken for the left side of the equation, and the center difference quotient was taken for the right side. The difference format at any node $\left(i,j,k,t\right)$ in the panel is

(16) $$\begin{array}{cc} \frac{h_{i,j,k}^{t+1}-h_{i,j,k}^{t}}{∆t} & =\frac{D_{i+\frac{1}{2},j,k}^{t}\cdot \left(h_{i+1,j,k}^{t}-h_{i,j,k}^{t}\right)-D_{i-\frac{1}{2},j,k}^{t}\cdot \left(h_{i,j,k,t}^{t}-h_{i-1,j,k}^{t}\right)}{∆x^{2}}\\ & +\frac{D_{i,j+\frac{1}{2},k}^{t}\cdot \left(h_{i,j+1,k}^{t}-h_{i,j,k}^{t}\right)-D_{i,j-\frac{1}{2},k}^{t}\cdot \left(h_{i,j,k}^{t}-h_{i,j-1,k}^{t}\right)}{∆y^{2}}\\ & +\frac{D_{i,j,k+\frac{1}{2}}^{t}\cdot \left(h_{i,j,k+1}^{t}-h_{i,j,k}^{t}\right)-D_{i,j,k-\frac{1}{2}}^{t}\cdot \left(h_{i,j,k}^{t}-h_{i,j,k-1}^{t}\right)}{∆z^{2}} \end{array}$$

Let $r_{1}=\frac{∆t}{∆x^{2}}$; $r_{2}=\frac{∆t}{∆y^{2}}$; $r_{3}=\frac{∆t}{∆z^{2}}$;

It can be simplified as

(17) $$\begin{array}{cc} h_{i,j,k}^{t+1} & =h_{i,j,k}^{t}+r_{1}\cdot D_{i-\frac{1}{2},j,k}^{t}\cdot h_{i-1,j,k}^{t}+\left[-r_{1}\cdot \left(D_{i-\frac{1}{2},j,k}^{t}+D_{i+\frac{1}{2},j,k}^{t}\right)\right]\cdot h_{i,j,k}^{t}+r_{1}D_{i+\frac{1}{2},j,k}^{t}h_{i+1,j,k}^{t}\\ & +r_{2}\cdot D_{i,j-\frac{1}{2},k}^{t}\cdot h_{i,j-1,k}^{t}+\left[-r_{2}\cdot \left(D_{i,j-\frac{1}{2},k}^{t}+D_{i,j+\frac{1}{2},k}^{t}\right)\right]h_{i,j,k}^{t}+r_{2}D_{i,j+\frac{1}{2},k}^{t}h_{i,j+1,k}^{t}\\ & +r_{3}\cdot D_{i,j,k-\frac{1}{2}}^{t}\cdot h_{i,j,k-1}^{t}+\left[-r_{3}\cdot \left(D_{i,j,k-\frac{1}{2}}^{t}+D_{i,j,k+\frac{1}{2}}^{t}\right)\right]\cdot h_{i,j,k}^{t}+r_{3}D_{i,j,k+\frac{1}{2}}^{t}h_{i,j,k+1}^{t} \end{array}$$

Some parameters in Equation (17) can be calculated using the following equations:

$$D_{i+\frac{1}{2},j,k}^{t}=\frac{1}{2}\left(D_{i+1,j,k}^{t}+D_{i,j,k}^{t}\right);\;D_{i-\frac{1}{2},j,k}^{t}=\frac{1}{2}\left(D_{i-1,j,k}^{t}+D_{i,j,k}^{t}\right)$$

$$D_{i,j+\frac{1}{2},k}^{t}=\frac{1}{2}\left(D_{i,j+1,k}^{t}+D_{i,j,k}^{t}\right);\;D_{i,j-\frac{1}{2},k}^{t}=\frac{1}{2}\left(D_{i,j-1,k}^{t}+D_{i,j,k}^{t}\right)$$

(18) $$D_{i,j,k+\frac{1}{2}}^{t}=\frac{1}{2}\left(D_{i,j,k+1}^{t}+D_{i,j,k}^{t}\right);\;D_{i,j,k-\frac{1}{2}}^{t}=\frac{1}{2}\left(D_{i,j,k-1}^{t}+D_{i,j,k}^{t}\right)$$

The relative humidity value of the internal units (3D diffusion) of the concrete pavement slab can be calculated using the differential format of Equation (17). The differential format for calculating the relative humidity of the slab corner (1D diffusion) and slab edge (2D diffusion) units can be obtained by multiplying the corresponding sub items in Equation (17) by 1/2.

### 2.2. Division of 3D Mesh

**Figure 2:**
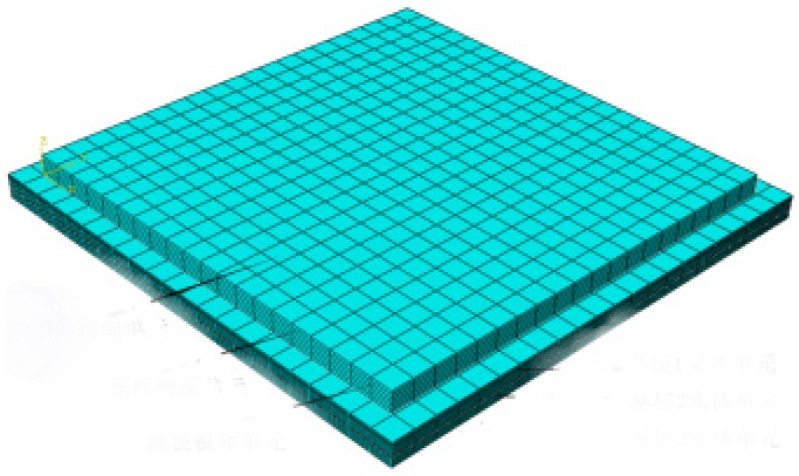
Schematic diagram of slab unit division.

**Figure 3:**
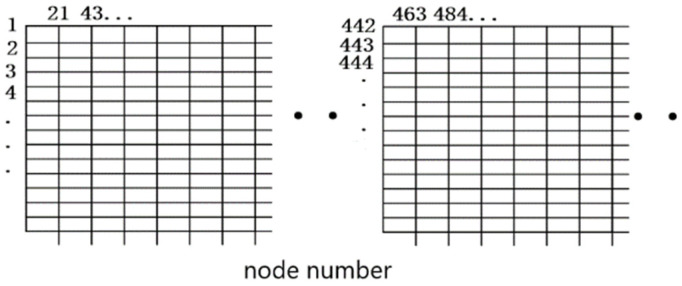
Schematic diagram of the slab node numbering.

The pavement slab was divided into 20 units along the length and width directions and was divided into 6 units along the depth direction by an integer multiple of 0.02 m. Taking the pavement slab with the size of 5 m × 4.5 m × 0.24 m as the example, there was a total of 2400 units with the size of 25 cm × 22.5 cm × 40 m, each depth plane containing 441 nodes, and the entire model had a total of seven layers, with a total of 3087 nodes. The specific mesh generation is shown in Figure 2 and Figure 3.

## 3. Early Stage Behavior of Pavement Environmental Field in Humid and Hot Areas

### 3.1. Working Condition Design

**Figure 4:**
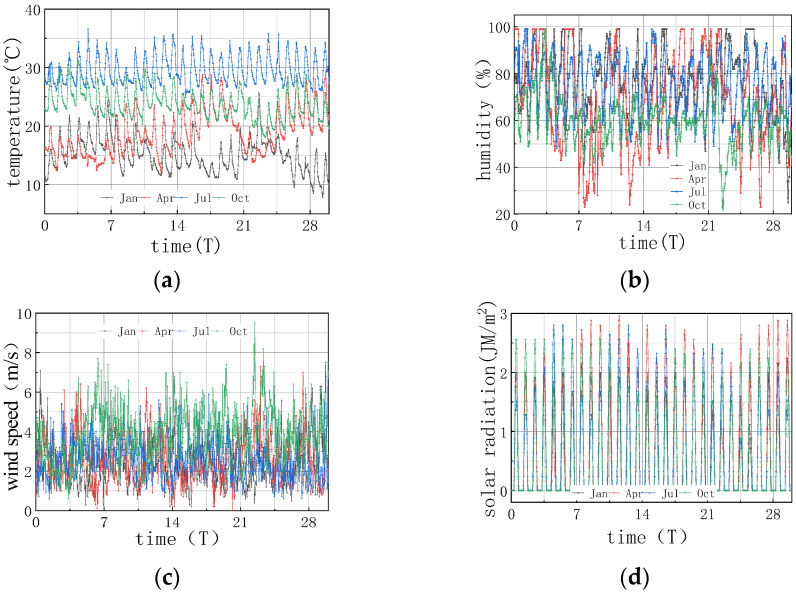
Measured values of environmental meteorology in Xiamen. (**a**) Measured value of environmental temperature in Xiamen. (**b**) Measured value of environmental humidity in Xiamen. (**c**) Measured value of windspeed in Xiamen. (**d**) Measured value of solar radiation in Xiamen.

For the pavement construction of a coastal airport in a humid and hot area, the typical summer weather of high temperature and humidity was selected as the reference condition, and the meteorological environment of July in Xiamen was selected, including environmental temperature, humidity, windspeed, and solar radiation intensity, as shown in Figure 4. The maintenance method under the reference working condition was watering maintenance with geotextile, and the detailed parameters are shown in Table 1. In order to compare different environmental parameters and conditions, in combination with the annual meteorological average in Xiamen, the environmental parameters in January, April, and October were selected as the comparison conditions for winter, spring, and autumn, respectively. The actual measured values of environmental and meteorological conditions for each month are shown in Figure 4.

It can be seen from Figure 4 that the overall summer climate in Xiamen was characterized with high temperature and high humidity, with a highest temperature of 36.6 °C and a lowest temperature of 24.9 °C; the diurnal temperature difference was not large due to the regulation of ocean temperature. The humidity in Xiamen varied greatly in July, with the highest value being 99% and the lowest being 48%. The summer windspeed and solar radiation in this area were relatively large, with a daily maximum solar radiation of 2.8 MJ/m^2^ and a windspeed of 7.5 m/s. The environmental temperature, humidity, windspeed, and solar radiation in January, April, July, and October all changed during day and night, with varying amplitudes, and the temperature changed prominently between the four seasons.

### 3.2. Distribution of 3D Humidity Field Properties of Concrete Pavement Slabs at Early Stage

**Figure 5:**
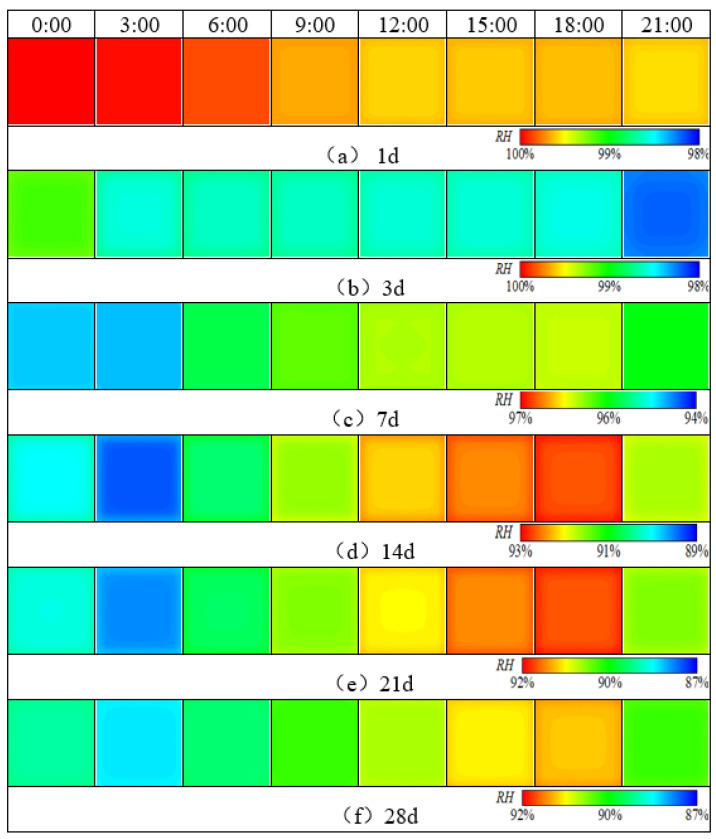
Humidity distribution at a distance of 4 cm from the top of the slab.

**Figure 6:**
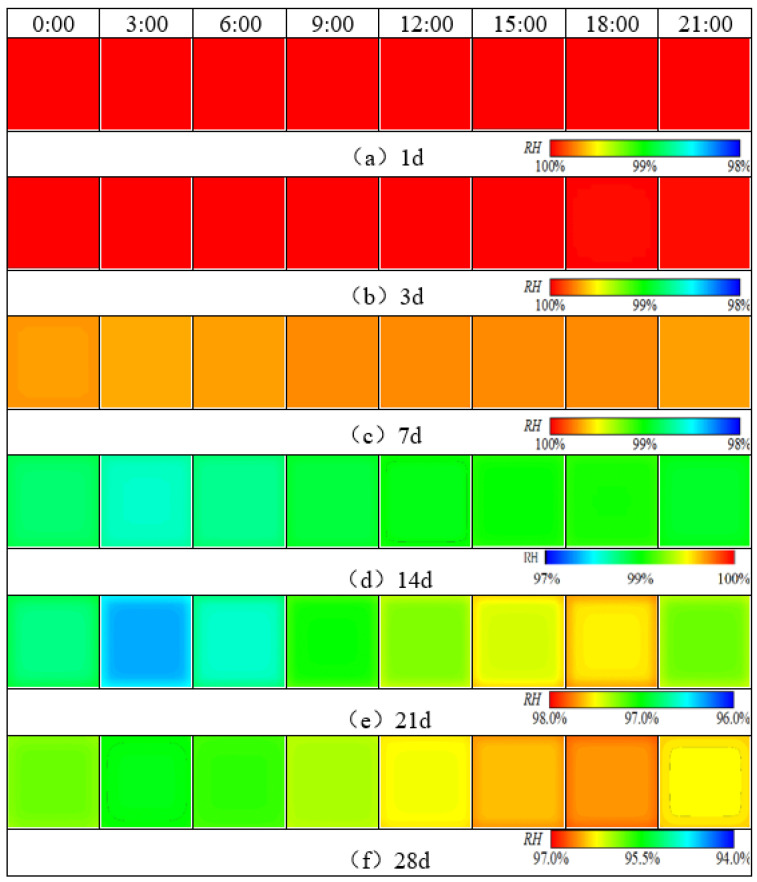
Humidity distribution at a distance of 8 cm from the top of the slab.

Based on the 3D humidity field simulation program for cement concrete pavements, the 3D evolution behavior of the humidity field of cement concrete pavement at early stage under the reference condition without maintenance during summer construction was calculated, and the results are shown in Figure 5 and Figure 6.

**Figure 7:**
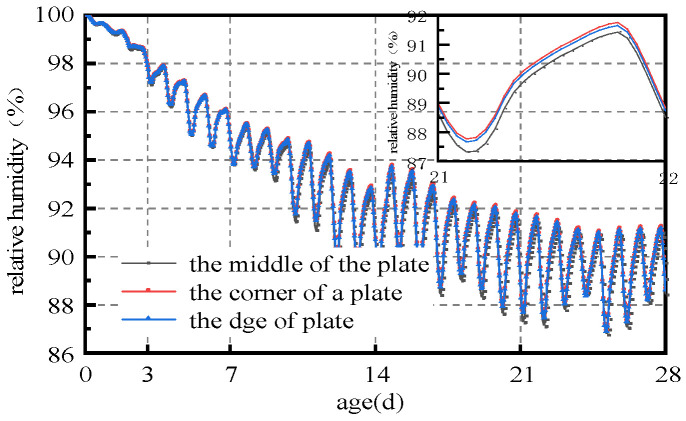
Typical humidity curve at 4 cm from the top of the slab.

From the above figure, it can be seen that the evolution behavior of the 3D humidity field of the cement concrete pavement of the coastal airport in the humid and hot region under the reference working condition environmental field at early stage mainly exhibited the following properties:

(1) As can be seen from Figure 5 and Figure 7, the humidity field in the first 3 days had a linear relationship with time, and the relative humidity of the slab gradually decreased with time. After 3 days, the humidity field changed periodically with the environmental field. The reason is that the internal hydration reaction of concrete in the first 3 days had a greater impact on the humidity field than that of the external environment. The variation in the humidity field mainly came from the hydration reaction, which consumed its own moisture and gradually reduced humidity. After 7 days, the humidity field was relatively low from 15:00 h to 21:00 h, and relatively high from 6:00 h to 12:00 h, indicating that the variation in the internal humidity field of the concrete after 7 days was dominated by the environmental field. The environment field was characterized by high temperature, low humidity, and large solar radiation from 11:00 h to 17:00 h, while from 0:00 h to 6:00 h it was characterized by low temperature, high humidity, and no solar radiation. However, the humidity field had a certain time lag compared with the environmental field.

(2) From the top to the bottom of the slab, the humidity field exhibited a significant difference, and the difference increased with time. The humidity of the slab surface at the distance of 4 cm from the top of the slab decreased from 100% to about 98.2% in the first 3 days, and the humidity of the slab surface at the distance of 8 cm from the top of the slab decreased from 100% to about 99.97%, and the relative humidity gradually decreased with time. The influence of the environmental field on the 3D humidity field inside the concrete gradually decreased along the depth direction. For the pavement concrete, the environmental field had the greatest impact at a distance of about 4 cm from the surface layer. This location was located at the boundary between the slurry extraction layer and the concrete layer, and the humidity stress caused by the humidity field tended to cause peeling damage.

(3) The humidity on the same plane slab was larger inside and smaller outside, and the dividing line gathered towards the middle of the slab with time. As can be seen from Figure 5, Figure 6 and Figure 7, the humidity in the slab was lower than the humidity at the slab corner, and slightly lower than the humidity at the slab edge. The humidity boundary gradually retracted from the edge to the center of the slab. The boundary was similar to the slab shape and diffused from 2 cm to 10 cm from the edge before entering a stable status. The reason is that the contacted surface between the slab boundaries and the external environment was larger than that in the middle of the slab, and the humidity diffusion was faster. The humidity boundary gradually diffused towards the slab until it reached an equilibrium state.

(4) Slab edges were more sensitive to the environment than the middle of the slab. From the analysis of (3), it can be concluded that when the external environment humidity was high, the humidity at the slab edge was higher than in the middle of the slab. Conversely, when the environmental humidity was low, the humidity at the slab edge was lower than in the middle of the slab.

### 3.3. Discussion and Analysis

From the above analysis, it could be deduced that the environmental field had an obvious influence on the distribution of 3D humidity field properties of the concrete pavement slabs at an early stage, which can be summarized as the following aspects:

(1) The environmental field had an obvious influence on the humidity of the surface layer of concrete slabs. The humidity of the slab surface changed periodically with the environment, but there was a certain lag compared to the environmental field. The humidity variation in the first 2 days fluctuated weakly with the environment, and then the fluctuation amplitude gradually increased until it was consistent with the environmental variations.

(2) The humidity gradient inside the concrete slab along the slab thickness direction was significant. The humidity difference between the top and bottom of the concrete slab was always negative, namely, the relative humidity gradually increased from the top to the bottom of the slab. This was ascribed to the fact that the bottom of the slab did not contact with the external environment and was generally in a saturated state, while the slab surface underwent real-time humidity exchange with the environment. Due to the periodic variation in the relative humidity at the top of the slab with the environmental field, the humidity difference between the bottom and top of the slab also exhibited periodic variation.

(3) The humidity gradient on the horizontal surface of the concrete slab was significant. The relative humidity of the slab panel gradually concentrated towards the center of the slab with time. The closer it was to the top of the slab, the more obvious the trend was. The humidity boundary was similar to the shape of the slab, and gradually retracted from the edge to the center of the slab until it reached balanced status.

(4) The sensitivity of the relative humidity of slab center, slab edge, and slab corner to the environment was slab center< slab edge< slab corner. Slab corner humidity was the most sensitive to the external environment, followed by slab edge, and the slab center. The reason is that there were three sides of the slab corner that were in contact with the external environment, which caused humidity exchange, making it easier to balance with the environmental humidity. Nevertheless, only one surface was in contact with the external environment, and the humidity diffusion speed was the slowest. Two surfaces of the slab were in contact with the external environment, and the humidity diffusion speed was between the slab center and the slab corner.

## 4. Effect of Different Factors on the Humidity Field of Concrete Pavement Slabs at an Early Stage

The middle part of the slab was taken as the analysis object; based on the 3D humidity simulation program for cement concrete pavement slabs, the effects of different factors on the evolution behavior of the 3D humidity field of concrete pavement slabs were studied based on the above working conditions.

### 4.1. Effect of Environmental Parameters

#### 4.1.1. Effect of Environmental Humidity

**Figure 8:**
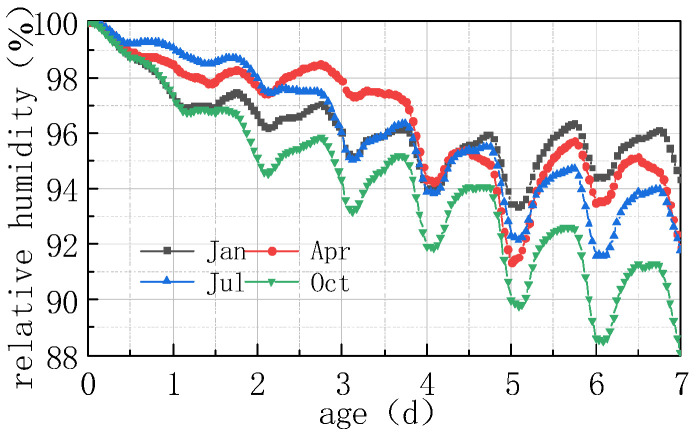
Effect of environmental humidity on RH.

**Figure 9:**
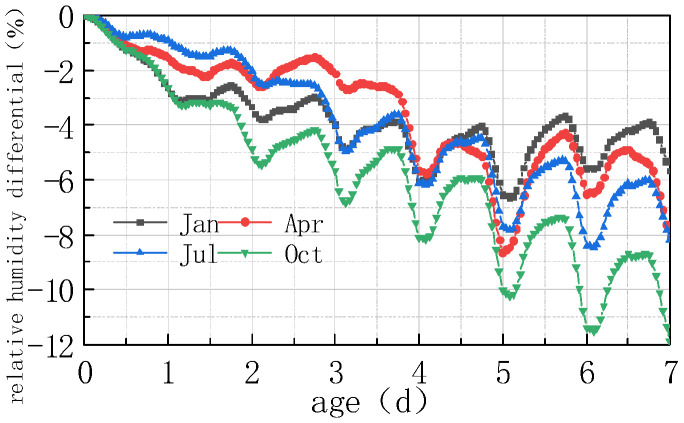
Effect of environmental humidity on ΔRH.

The impact of different humidity environments on the relative humidity (RH) at the top of the concrete pavement slab and the humidity difference (ΔRH) between the bottom and the top of the slab was analyzed. The calculation results were shown in Figure 8 and Figure 9.

**Figure 10:**
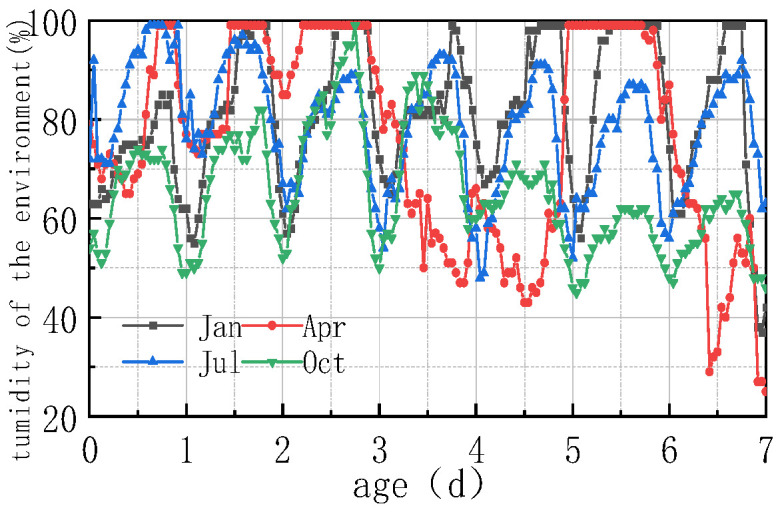
Environmental humidity in different months.

As can be seen from Figure 8 and Figure 9, the overall RH in the top of the pavement slab exhibited a downward trend. The curve peaks and valleys in the first 2 days were not obvious, and then RH showed a cyclical fluctuation trend with daily humidity variations, the variation trend of RH and ΔRH exhibited a linear relationship. It can be deduced that the laws include the following points:

(1) The environmental humidity field had a significant impact on the humidity field of the pavement. Under different humidity conditions, the relative humidity value in January at 7 days was the highest, with an RH of 94.328%. The RH in April was similar to that in July, reaching approximately 91%, and the relative humidity value in October was the lowest, with an RH of 88.128%. The relative humidity difference in January was the smallest, with the absolute value of ΔRH being 5.672%. The relative humidity difference was the largest in October, with the absolute value of ΔRH being 11.872%. The results were consistent with the monthly humidity laws in Figure 10.

(2) The relative humidity curve in the slab was consistent with the law of the humidity field and fluctuated in a daily cycle. It can be seen from Figure 8, Figure 9 and Figure 10 that the environmental humidity curves in July and October were relatively stable, presenting a daily sinusoidal fluctuation. The corresponding RH and ΔRH curves also presented the same sinusoidal fluctuations, and the environmental humidity stability was in line with the environmental humidity curve.

(3) Environmental field fluctuations had a significant impact on the humidity of the slab. After finishing the pouring of the concrete slab, the top of the slab was in direct contact with the external environment, resulting in a humidity difference between the slab surface and the environmental field, and therefore causing the relative humidity in the slab to gradually decrease from 100% with time. In Figure 8 and Figure 9, a horizontal section and a sharp decline section could be found in the curve of RH and ΔRH in April at 2–4 days due to the fact that the environmental humidity field exhibited a large humidity value and a small variation amplitude at 2–3 days, and a sharp decrease in humidity at 3–4 days. The results indicated that the humidity field of the concrete pavement slab was consistent with the law of the environmental humidity field and had a certain lag. This law was in accordance with the 3D humidity field characteristics of the concrete pavement slab at the early stage analyzed above.

(4) The variations in relative humidity in the first 2 days were caused by both cement hydration and environmental humidity, and then they were mainly affected by environmental humidity field. The relative humidity curve in the slab top over the previous 2 days decreased significantly, and the sinusoidal rule was not obvious because of the intense hydration reaction from the previous 2 days and the consistent consumption of internal moisture. The curve of relative humidity reflected the duplicate effect of hydration consumption humidity and moisture exchange. After 2 days, hydration was basically completed, and the relative humidity curve mainly reflected the humidity exchange effect with environmental humidity.

#### 4.1.2. Effect of Windspeed

**Figure 11:**
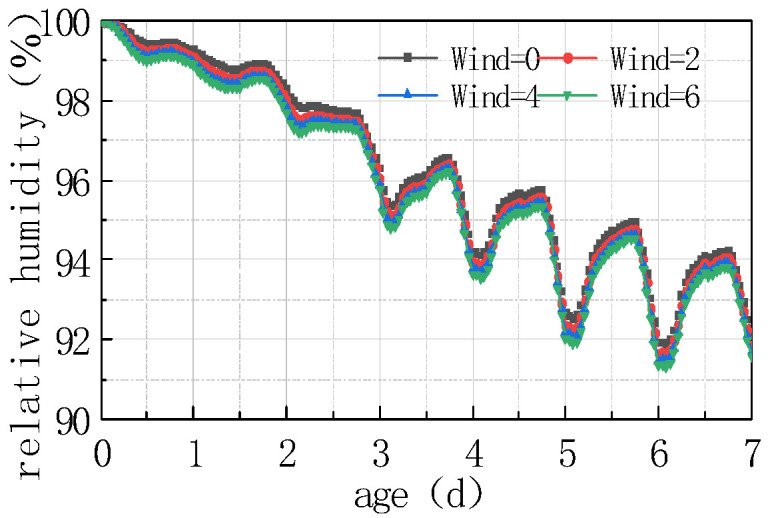
Influence of windspeed on RH.

**Figure 12:**
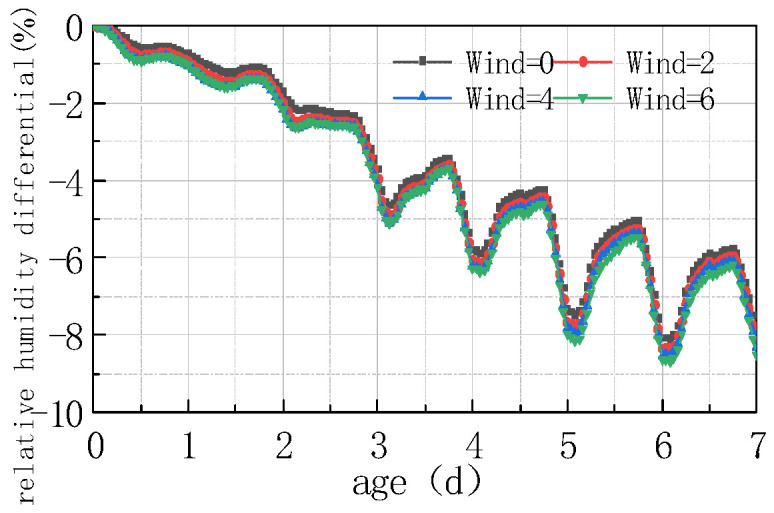
Influence of windspeed on ΔRH.

See Figure 11 and Figure 12 for the relative humidity difference between the top and bottom of the concrete slab under different windspeeds.

As can be seen from the figure, the relative humidity decreased with windspeed, and the humidity difference increased with windspeed, but the amplitude of decrease and increase were relatively small. At 7 days, the windspeed increased from 2 m/s to 6 m/s, and the relative humidity at the top and in the middle of the slab only decreased by 0.593%, while the humidity difference increased by 0.59%. The reason is that airflow facilitated moisture evaporation, and the greater the windspeed, the faster the moisture evaporation occurs. Meanwhile, this also accelerated the moisture exchange between the air and the slab. In coastal environments, the effect of windspeed on air humidity was not prominent. When the slab humidity and environmental humidity were in balance, the water exchange reached an equilibrium state. The declining rate of concrete humidity was determined by the concrete humidity diffusion coefficients; thus, the influence of windspeed was not significant.

#### 4.1.3. Effects of Solar Radiation

**Figure 13:**
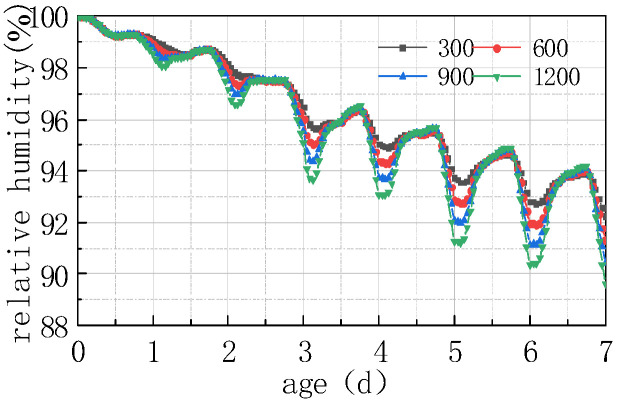
Impact of solar radiation on RH.

**Figure 14:**
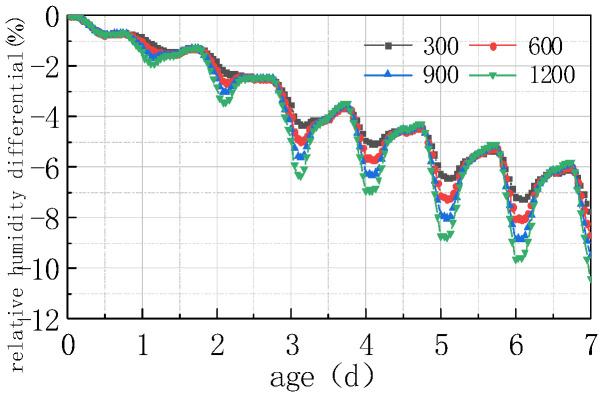
Impact of solar radiation on ΔRH.

Shown in Figure 13 and Figure 14 are the curves of the relative humidity at the top of the concrete slab and the humidity difference between the top and the bottom of the slab, respectively, at solar radiations of 300 W/m^2^, 600 W/m^2^, 900 W/m^2^, and 1200 W/m^2^.

As can be seen from the above figure, solar radiation had a significant influence on the humidity value of the concrete slabs. The greater the solar radiation value, the greater the fluctuation in the relative humidity and humidity difference curves. The curve of the first 3 days was mainly affected by the internal hydration of the concrete, and the impact of environmental factors on its fluctuating amplitude was relatively slight. After 3 days, the fluctuation amplitude gradually increased, and the maximum relative humidity difference reached 2.62% at 7 days. The reason is that the increase in solar radiation leads to an increase in the temperature of the concrete slab, but a decrease in the saturated vapor pressure inside the concrete, thereby leading to a decrease in the saturated humidity inside the concrete, manifested as a decrease in the relative humidity value of the concrete slab. The results of this study are somewhat in compliance with those reported by Nasir et al. [28] on hot weather climatic conditions during the months of July to August in a Saudi Arabian region. They studied the strength, pulse velocity, water absorption, drying shrinkage, and plastic shrinkage properties and found that the temperature gradient between the concrete and ambient environment plays vital role in mitigating the problems associated with the harsh weather. They also recommended following the guidelines of ACI Committee 305 for hot weather concreting.

### 4.2. Effect of Material Parameters

#### 4.2.1. Effect of Cement Type

**Figure 15:**
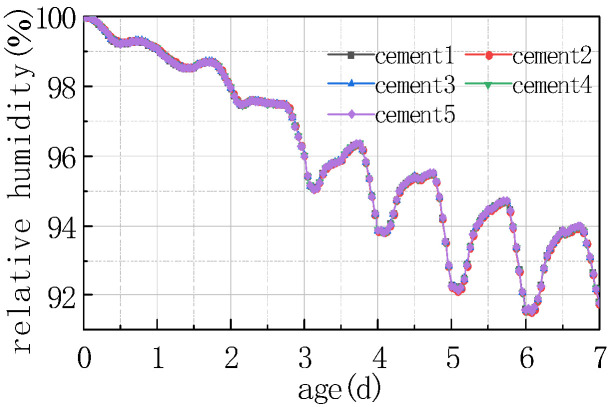
Effect of cement on RH.

**Figure 16:**
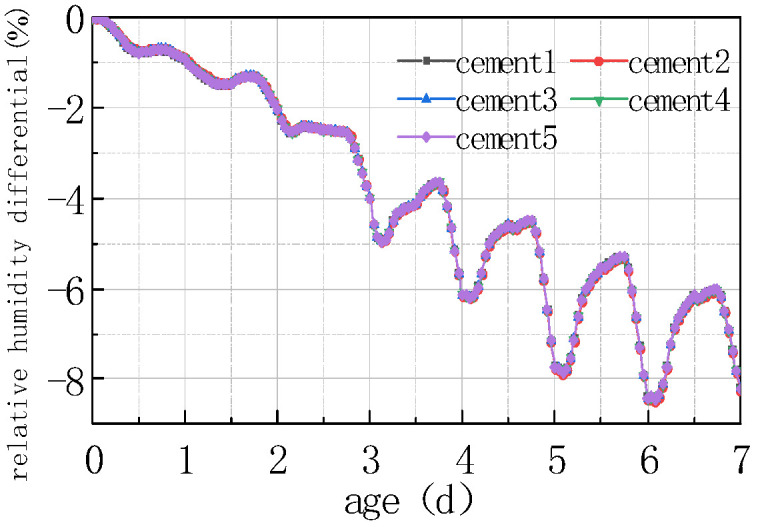
Effect of cement on ΔRH.

Five different types of Portland cement were selected, and their performance on the relative humidity at the top of the concrete slab and the humidity difference between the bottom and the top of the slab were also calculated. The results are shown in Figure 15 and Figure 16.

Based on the above figure, it can be observed that the cement component had little impact on the relative humidity of the top of the concrete pavement slab, and the relative humidity difference between the concrete slabs of different types of cement was less than 0.5%. Due to the humidity exchange between the slab surface and the external environment, the humidity level at the bottom of the slab was greater than at the top of the slab, while the humidity difference between the bottom and the top of different cement concrete slabs was less than 0.5%.

The influence of cement type on the humidity field of the concrete pavement slabs was mainly reflected in the aspect of heat release from cement hydration. Nevertheless, cement hydration mainly occurred before concrete setting, at which time the relative humidity value inside the concrete was relatively high and generally in a saturated state. The temperature difference generated by the heat release from the hydration of different types of cement on the inside of the concrete was insufficient to exert an obvious impact on the relative humidity, namely, there was a phenomenon that the relative humidity curves of different types of cement concrete pavement slabs basically overlapped.

#### 4.2.2. Effect of Water–Cement Ratio

**Figure 17:**
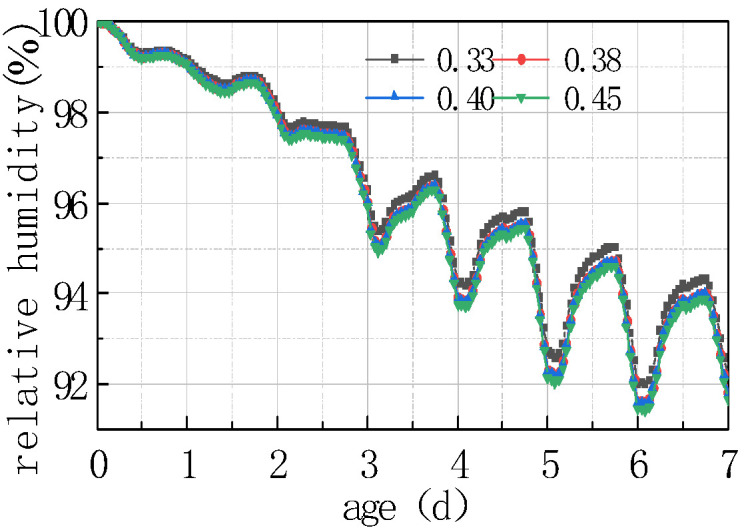
Effect of water–cement ratio on RH.

**Figure 18:**
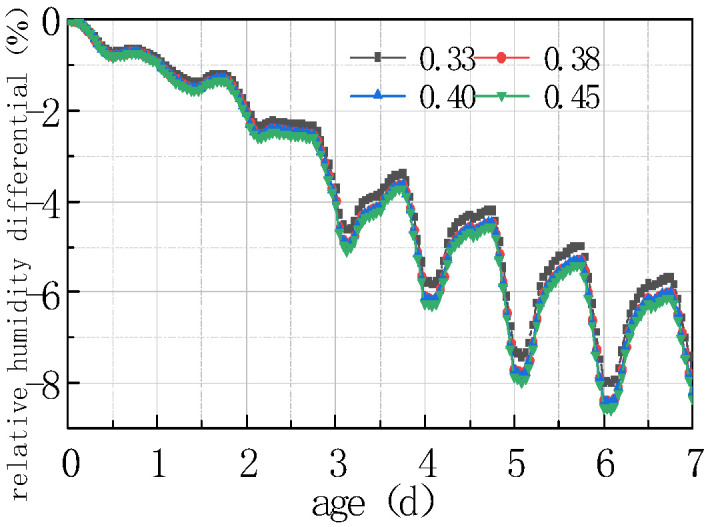
Effect of water–cement ratio on ΔRH.

Shown in Figure 17 and Figure 18 is the relative humidity difference between the top and bottom of the concrete slabs under different water–cement ratios.

It can be seen from the above figure that the relative humidity at the top of the concrete slab decreased with the water–cement ratio, and the relative humidity difference between different water–cement ratios was more significant with time. The law of the relative humidity difference between the bottom and the top of the slab was the same as the relative humidity at the slab top. The reason is that the increase in water–cement ratio led to an increase in free water content in the concrete, thereby accelerating the hydration of the cement, as well as enhancing the heat generated by hydration, resulting in an increase in the internal temperature of the concrete, and accelerating the evaporation of water. The combined action of the two effects would reduce the humidity on the top of the concrete slab. The curve after 2 days mainly reflected the humidity exchange between the concrete slab and the external environment. The lower the relative humidity of the slab top was, the more obvious the humidity exchange was. The curve reflected that the relative humidity of the slab top varied periodically with environmental humidity. The relative humidity difference between the peaks and troughs of different water–cement ratios slightly increased with time, but the amplitude was not significant. The findings of this study are in agreement with those reported by Khan et al. [29] wherein the impact of traditional and five types of pozzolanic materials were explored under hot weather conditions. They revealed that some pozzolanic materials including fly ash, ground granulated blast-furnace slag, Superpozz, and natural pozzolan when blended in optimum dosage with Portland cement enhance the early stage and later stage characteristics of concrete due to their slow pozzolanic reactivity in the early period, which prevents the formation of sudden and nonuniform gel products, making them ideal candidates for aggressive environmental conditions. Further, they also emphasized that proper field curing, including wet burlap and application of one-time curing compound, results in a durable concrete.

### 4.3. Effect of Structure and Construction Parameters

#### 4.3.1. Effect of Thickness of the Pavement Slab

**Figure 19:**
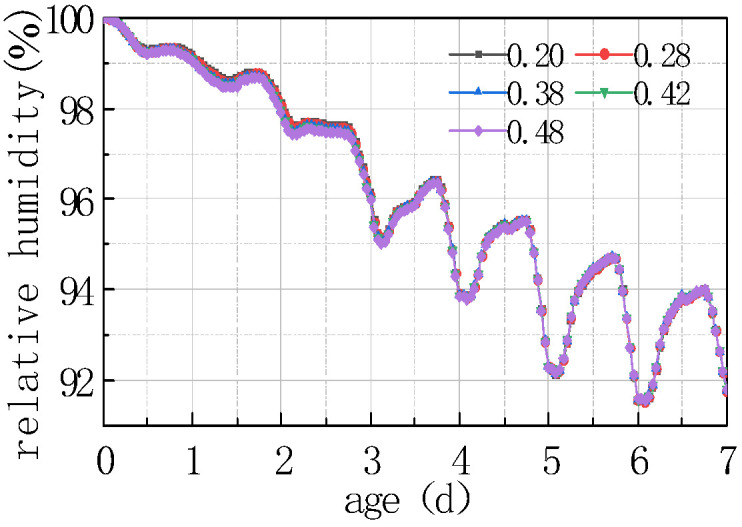
Effect of slab thickness on RH.

**Figure 20:**
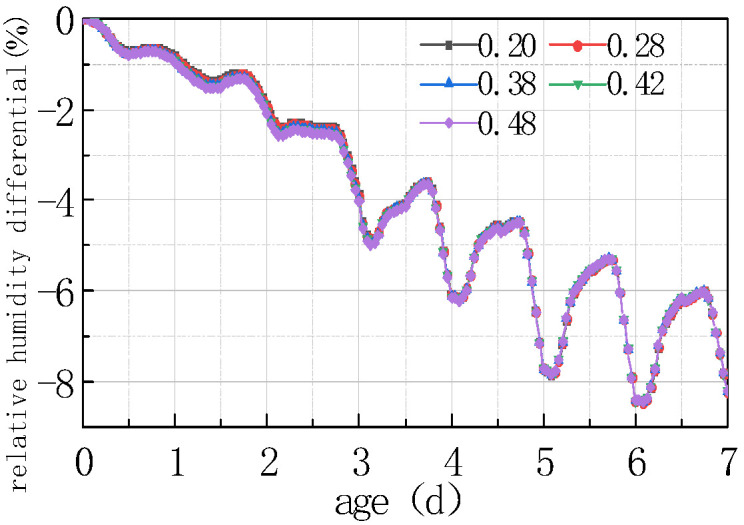
Effect of slab thickness on ΔRH.

The effect of pavement slab thickness on relative humidity at the top of the slab and the humidity difference between the bottom and the top of the slab were analyzed. The calculation results are shown in Figure 19 and Figure 20.

It can be seen from Figure 19 and Figure 20 that the thickness of the concrete slab had no obvious impact on the relative humidity at the top of the slab and the humidity difference between the bottom and the top of the slab. The difference in the relative humidity at the tops of slabs with different thicknesses was not greater than 0.5%, and the relative humidity at a distance of 2 cm from the top of the slab was basically not affected by slab thickness.

#### 4.3.2. Effect of Paving Time

**Figure 21:**
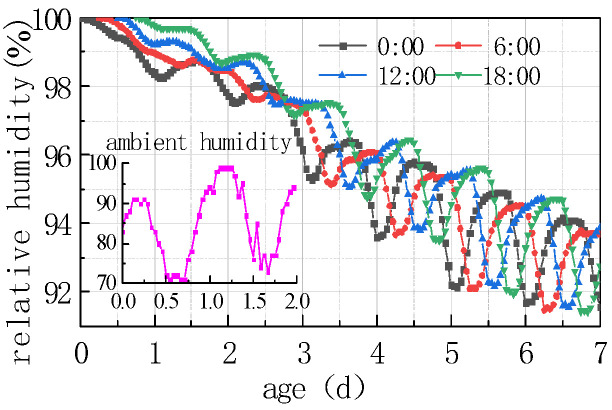
Effect of paving time on RH.

**Figure 22:**
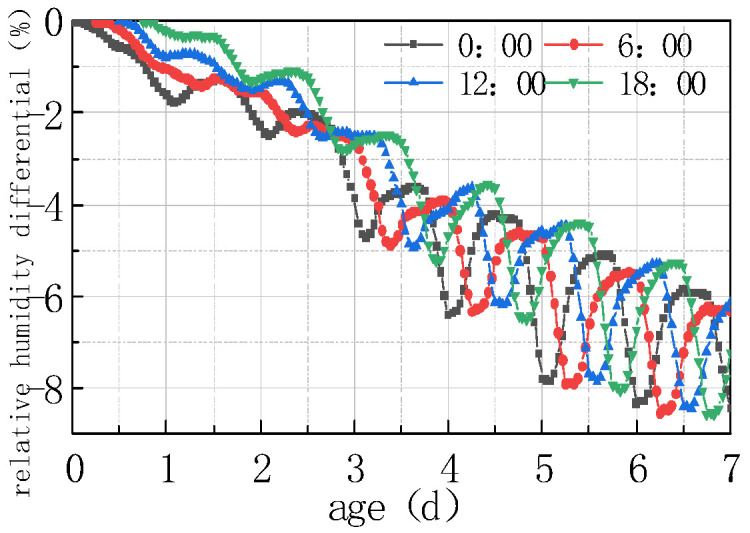
Effect of paving time on ΔRH.

**Figure 23:**
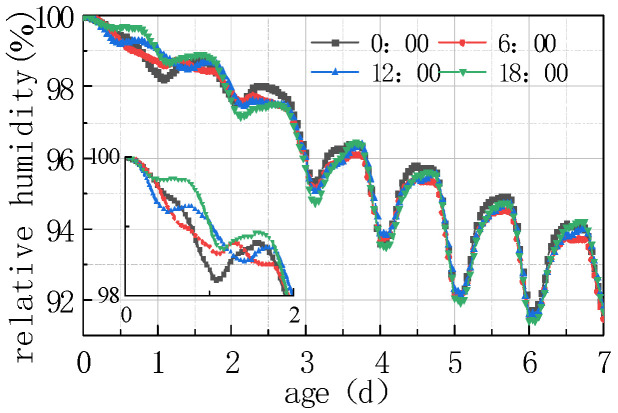
Comparison diagram of impact of paving Time on RH.

**Figure 24:**
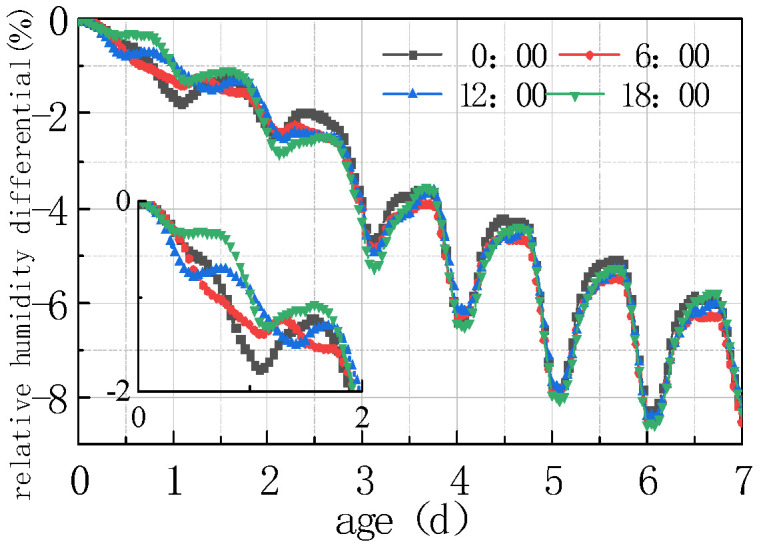
Comparison diagram of impact of paving Time on ΔRH.

Due to the periodic variation in daily meteorological data and significant differences between peak and trough values, paving concrete pavement at different times exposes the pavement slabs to different initial environmental fields, which inevitably exerted a certain impact on the humidity diffusion of the pavement slabs. To analyze the influencing rule, concrete pouring was conducted at four times, viz. 0:00 h, 6:00 h, 12:00 h, and 18:00 h. The calculation results are shown in Figure 21, Figure 22, Figure 23 and Figure 24.

According to the analysis in Figure 21, Figure 22, Figure 23 and Figure 24, the relative humidity at the top of the concrete pavement slab exhibited a significant difference in the first 48 h, and the difference gradually weakened as time went on. The characteristics of early stage relative humidity and the humidity difference curve included the following aspects:

(1) The fluctuation laws of the relative humidity curve at the top of the concrete slab at different paving times was the same, and the difference between the peaks and troughs on the time axis was the same as the difference at the paving time, that is, the relative humidity curve at the top of each slab fluctuated cyclically for 24 h from paving time.

(2) After 24 h of paving at different times, the humidity at the top of the slab showed a downward trend, with the maximum decline in the relative humidity curve occurring when paving at 00:00, with an amplitude of 1.541%. At 18:00, the decreasing trend of the paving relative humidity curve was the smallest, with an amplitude of 1.189%.

Compared to the environmental humidity in Figure 21, after the completion of pouring at the time of 0, the slab humidity was in a saturated state, which was much higher than environmental humidity. Humidity exchange occurred between the slab and the environment, the relative humidity decreased, and then there was a large decline in environmental humidity, as well as a decline in the relative humidity of the slab. In addition, there was a lag in the relative humidity of the slab compared to the environmental humidity; the decreasing amplitude of the relative curve at the top of the poured concrete slab was the largest at 00:00. After the completion of pouring at 18:00, environmental humidity appeared to rise for about 12 h, while the relative humidity curve at the top of the slab decreased relatively gently during this period. With the subsequent lag effect, the sharp decline in the first 24 h only remained for about 6 h, and the decline amplitude of the relative humidity curve when pouring at 18:00 was the smallest. From the above analysis, it can be seen that concrete pouring around 18:00 has the smallest impact on the internal humidity changes of the concrete, so the stress generated by humidity changes is also the smallest.

(3) The relative humidity curve at the top of the slab at different pouring times gradually increased with time. As can be seen from Figure 21, in the first 7 days, the superposition of peaks and troughs of each curve occurred on the sixth day, with the maximum difference being 3.466% of the curve difference between 18:00 and 06:00, which was the same with the peak and trough time of the humidity curve.

(4) Figure 23 and Figure 24 show the comparison curves of the same time length after pouring at different times. The variation rules of the relative humidity and humidity difference curves of the concrete slab in the first 2 days were inconsistent, and then the curves gradually overlapped, exhibiting the same rules. This indicated that the influence of environmental factors on the humidity of the concrete slabs poured at different times was concentrated in the first 60 h.

#### 4.3.3. Effect of Maintenance Methods

**Figure 25:**
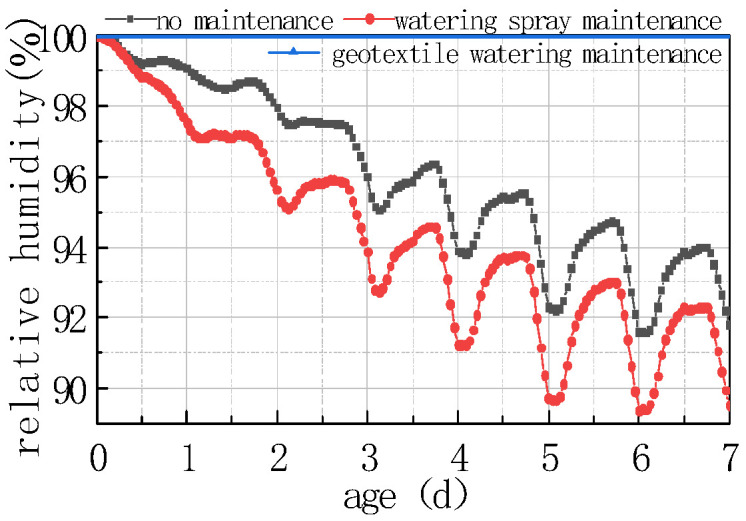
Effect of curing methods on RH.

**Figure 26:**
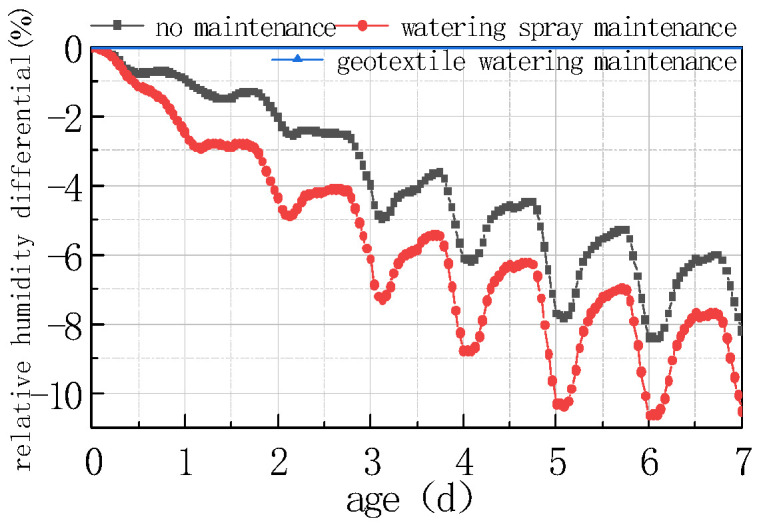
Effect of curing methods on ΔRH.

Three typical curing methods, i.e., no curing, water curing, and geotextile + water curing, were used to explore the impact of curing methods on the humidity field of the concrete slabs. The environmental humidity of the water curing was proposed to be maintained at 70%, and the environmental humidity of the geotextile + water curing was proposed to be maintained in a saturated state. The calculation results are shown in Figure 25 and Figure 26.

As can be seen from the figure, the curing method had a significant impact on the humidity field at the top of the concrete slab and the humidity difference between the bottom and the top of the slab; the details are listed as follows:

(1) Except for the environmental humidity of 100%, the relative humidity curve at the top of the slab and the humidity difference curve at the bottom and the top of the slab possessed the same fluctuation rules, which fluctuated periodically with the environment for 24 h. This indicated that the humidity field was closely related to environmental temperature, windspeed, and solar radiation, in addition to being affected by environmental humidity, and was represented by the combined effect of various factors.

(2) During water curing, the relative humidity value at each time point on the top of the concrete slab was greater than that with no curing, and the difference between the peaks and troughs of the relative humidity curve on the top of the concrete slab with water curing was smaller than with no curing.

(3) During geotextile + water curing, the relative humidity of each part inside the concrete slab was 100%, which was the most ideal state for concrete strength growth and internal stress.

From the above analysis, it can be seen that if no maintenance measures are taken when the concrete pavement is poured, the humidity of the slab dramatically decreases, resulting in a large humidity difference inside the slab, and forming a stress gradient that induces early stage cracking. As a result, a reasonable curing method is an important measure to ensure strength growth and other properties, and to prevent microcracks and cracks in the concrete.

## 5. Parameter Sensitivity Analysis

There are many factors that affect the 3D humidity field behavior of concrete pavement slabs. To study the sensitivity of each factor, a single factor was altered while ensuring that other parameters remained unchanged, and the sensitivity of each single factor to the concrete’s humidity field behavior was also analyzed separately.

Single-factor sensitivity was divided into three levels: high (S ≥ 2%), medium (1 ≤ S < 2), and low (S < 1%). According to the numerical simulation program for the 3D humidity field of the cement concrete pavement, the sensitivity of each factor is shown in Table 2.

As can be seen from Table 2, material parameters and structural parameters had a small impact on the 3D humidity field of the concrete pavement slab. Changing the cement type, water–cement ratio, and slab thickness did not cause more than 1% difference in the relative humidity at the top of the concrete slab or in the humidity difference between the bottom and the top, indicating that the impact of material and structural parameters on the humidity field can be ignored during the design process, and the stability and improvement of other performance aspects should be enhanced. Environmental parameters and construction parameters had a certain influence on the 3D humidity field of the concrete pavement slabs, with solar radiation and paving time having a weak influence. The sensitivity of windspeed to the 3D humidity field of the concrete slabs was at a middle level. The higher the windspeed, the lower the relative humidity at the top of the slab, and the greater the humidity difference between the bottom and the top of the slab. Environmental humidity and curing methods were highly sensitive to the 3D humidity field of the concrete slabs, especially the curing method, which was artificially controllable. Choosing an appropriate curing method was crucial to reducing early stage defects in concrete structures.

From the perspective of influencing characteristics, the influence of various parameters on the 3D humidity field of the concrete pavement slabs was mainly divided into two aspects, namely, direct influence and indirect influence.

(1) Direct influence refers to the variation in relative humidity value through the internal moisture of the concrete slab. The curing method, environmental humidity, environmental temperature, windspeed, cement type, and water–cement ratio all affected the humidity field by altering the moisture of the concrete slab. Among them, environmental humidity and curing methods influenced the humidity field by moisture exchange with the concrete slabs, environmental temperature and windspeed affected the humidity field by diffusing moisture from the concrete slabs, while the cement type and water–cement ratio affected the humidity field by absorbing moisture through chemical reactions.

(2) Indirect influence refers to the variations in relative humidity values through the saturated vapor pressure inside the concrete slab. Environmental temperature, solar radiation, cement type, and water–cement ratio all affected the saturated vapor pressure inside the concrete slab by changing the temperature of the slab. The indirect influence was generally weak and only had a fluctuating performance on the relative humidity of the slab but had little impact on the internal moisture content of the concrete within a certain period of time.

## 6. Conclusions

(1) The environmental field had a significant impact on the humidity of the surface of the concrete slabs. After the pouring of the concrete panel is completed, the humidity at 2 cm in the middle of the panel, 2 cm~6 cm at the corner, and 1/2 of the edge of the panel decreases to the lowest stage value, and the trend of humidity change basically coincides with the fluctuation trend of environmental humidity.

(2) The sensitivity of the surface humidity of concrete panels to the environment varies significantly. The order of sensitivity to the environment is board corner, board edge 1/2, and board center 2 cm.

(3) The humidity gradient inside the concrete panel is significant along the thickness direction, and at the same time, the humidity of the road panel concentrates towards the middle and bottom of the board. The difference between the humidity boundaries of 2 cm, 4 cm, and 6 cm from the top of the board and the maximum humidities in the slabs during the first 28 days are 1.3%, 2.3%, and 2.9%, respectively.

(4) Environmental parameters, construction parameters, and material and structural parameters all affected slab humidity through humidity exchange or by changing the saturated vapor pressure inside the slab. Environmental humidity and curing methods were highly sensitive to the humidity field of the concrete slabs, followed by windspeed. Solar radiation, cement type, water–cement ratio, and thickness were less sensitive to the humidity fields of the concrete slabs. During construction, hot seasons should be avoided; the periods should be increased to pour concrete in a selected ambient humidity. Moreover, appropriate curing methods should be adopted to reduce the humidity stress of slabs, eliminate early stage defects, and thereby improve their service life.

## Figures and Tables

**Table 1:** Working conditions and parameter values of 3D humidity field of panel.

Variable Type	Name of Parameter	Value Range	Reference Value
Mixture	Cement type/ASTM	Type Ⅰ–Ⅴ	Type I
Composition variables	Water–cement ratio	0.38~0.42	0.40
Environmental parameters	Daily average temperature/°C	Meteorological and weather measurements for Xiamen in January, April, July, and October	July
	Daily average humidity/°C		
	Environmental humidity/%		
	Daily average windspeed/m/s	0~6	3
	Daily maximum solar radiation intensity/w/m^2^	0~1200	600
	Sunlight duration/h	12	
Construction parameters	Thickness of pavement slab/cm	28~42	38
	Paving time/24 h	0~24	12
	Maintenance situation	With/without	Without

**Table 2:** Sensitivity of various parameters to the 3D humidity field of concrete slabs.

Parameter Situation	Working Condition Value	Reference Condition	Relative Humidity of the Slab/%			Humidity Difference between the Bottom and the Top of the Slab/%		
			Peak Value/%	The Difference with Reference Value /%	Sensitivity Level	Peak Value/%	The Difference with Reference Value/%	Sensitivity Level
Cement type	Type II	Type I	91.51	−0.05	Low	8.49	0.05	Low
	Type III		91.54	−0.02		8.46	0.02	
	Type IV		91.54	−0.02		8.46	0.02	
	Type V		91.55	−0.01		8.45	0.01	
Water–cement ratio	0.33	0.38	92	0.44	Low	8	−0.44	Low
	0.41		91.59	0.03		8.41	−0.03	
	0.45		91.43	−0.13		8.58	0.14	
Environmental humidity	January	July	94.33	2.77	High	5.61	−2.83	High
	April		91.89	0.33		7.63	−0.81	
	October		88.13	−3.43		11.5	3.06	
Windspeed	0 m/s	2 m/s	92.22	0.91	Middle	7.78	−0.91	Middle
	4 m/s		90.45	−0.86		9.55	0.86	
	6 m/s		89.6	−1.71		10.4	1.71	
Solar radiation	300 W/m^2^	600 W/m^2^	91.9	0.26	Low	8.11	−0.26	Low
	900 W/m^2^		91.44	−0.2		8.56	0.19	
	1200 W/m^2^		91.3	−0.34		8.7	0.33	
Slab thickness	0.2 m	0.42 m	91.52	−0.04	Low	8.48	0.04	Low
	0.28 m		91.54	−0.02		8.46	0.02	
	0.38 m		91.55	−0.01		8.45	0.01	
	0.48 m		91.56	0		8.44	0	
Paving time	0:00 h	13 h	91.55	−0.01	Low	8.45	0.01	Low
	6:00 h		91.46	−0.1		8.54	0.1	
	18:00 h		91.41	−0.15		8.59	0.15	
Maintenance method	Watering	None	89.39	−2.17	High	10.61	2.17	High
	Geotextile + watering		100	8.44		0	−8.44	

## Data Availability

Not applicable.
